# Tumour M2-PK as a stool marker for colorectal cancer: comparative analysis in a large sample of unselected older adults *vs* colorectal cancer patients

**DOI:** 10.1038/sj.bjc.6603712

**Published:** 2007-04-03

**Authors:** U Haug, D Rothenbacher, M N Wente, C M Seiler, C Stegmaier, H Brenner

**Affiliations:** 1 Division of Clinical Epidemiology and Aging Research, German Cancer Research Center; Bergheimer Street 20, 69115 Heidelberg, Germany; 2 Department of General, Visceral and Trauma Surgery, University of Heidelberg, Im Neuenheimer Feld 110, 69120 Heidelberg, Germany; 3 Saarland Cancer Registry, Virchowstreet 7, 66119 Saarbrücken, Germany

**Keywords:** colorectal cancer, diagnosis, stool test, tumour M2-PK

## Abstract

Stool testing based on tumour-derived markers might offer a promising approach for non-invasive colorectal cancer (CRC) screening. The aim of this study was to estimate the potential of a new test for faecal tumour M2-PK to discriminate patients with CRC from a large sample of unselected older adults. Faecal tumour M2-PK concentrations were determined in 65 CRC patients and in a population-based sample of 917 older adults (median age: 65 and 62 years, respectively). Sensitivity and specificity of the test were calculated at different cutoff values, and receiver-operating characteristic curves (ROC) were constructed to visualise the discriminatory power of the test. The median (interquartile range) faecal tumour M2-PK concentration was 8.6 U ml^−1^ (2.8–18.0) among CRC patients and <2 U ml^−1^ (<2–3.2; *P*<0.0001) in the population sample. At a cutoff value of 4 U ml^−1^, sensitivity (95% confidence interval) was 85% (65–96%) for colon cancer and 56% (41–74%) for rectum cancer. Specificity (95% confidence interval) was estimated to be 79% (76–81%). Given the comparatively high sensitivity of the tumour M2-PK stool test (especially for colon cancer) and its simple analysis, the potential use of the test for early detection of CRC merits further investigation. Possibilities to enhance specificity of the test should be explored.

Colorectal cancer (CRC) is one of the leading causes of cancer-related morbidity and mortality worldwide (Ferlay *et al*, 2004). Even in countries where up-to-date therapeutic options are available, more than 40% of CRC patients still die from the disease within 5 years after diagnosis (Brenner, 2002; Brenner *et al*, 2005). As survival is considerably better for early, localised CRC than for later stages and CRC might be prevented altogether by detection and removal of precancerous lesions, enhanced screening will be of crucial importance for further progress in reducing the burden of the disease.

Experience with faecal occult blood testing (FOBT) has shown the possibility of reducing both incidence and mortality owing to screening based on stool tests (Hardcastle *et al*, 1996; Mandel *et al*, 1999, 2000) and the advantages of this screening modality regarding acceptability and practicality compared with invasive screening options. However, given the limitations of FOBT, mainly the inherently low sensitivity to detect precancerous or cancerous lesions, there is still need for improvement. Therefore, the development of stool tests with better performance characteristics has become a focus of current research, both in the field of DNA-based stool markers and in the field of protein-based stool markers. For some of the new tests, preliminary results regarding sensitivity and specificity are encouraging but evidence of the performance of the new methods is mostly restricted to small-scale investigations in the clinical setting (Haug and Brenner, 2005a).

A novel approach based on the detection of proteins in stool derived from neoplastic colonocytes is the quantitative measurement of faecal tumour M2-pyruvate kinase (tumour M2-PK). Tumour M2-PK, an isoform of the glycolytic enzyme pyruvate kinase, is found in proliferating tissues with a high capacity for nucleic acid synthesis such as tumour cells (Mazurek *et al*, 2002a, 2002b). The aim of this study was to estimate the potential of testing for faecal tumour M2-PK to discriminate patients with CRC from a large sample of unselected older adults.

## MATERIALS AND METHODS

### Study population

Stool samples were collected from 65 CRC patients before cathartic preparation for surgery at the Department of Surgery, University Hospital of Heidelberg, Germany. Samples from CRC patients were collected between January 2003 and February 2005 and immediately frozen until laboratory analysis, which was performed in March 2005. Data concerning age, gender, body mass index, current smoking status, tumour stage, tumour location and neoadjuvant therapy were abstracted from the hospital charts.

For comparison, stool samples were analysed from 917 participants aged 50–75 years of a large-scale population-based cohort study (ESTHER=‘Epidemiologische Studie zu Chancen der Verhütung, Früherkennung und optimierten Therapie chronischer Erkrankungen in der älteren Bevölkerung’), aimed to evaluate new approaches to the prevention, early detection and therapy of chronic diseases among older adults. ESTHER is conducted in Saarland, a state located in SouthWest Germany, and recruitment took place between June 2000 and December 2002. Details of the study design have been reported elsewhere (Rothenbacher *et al*, 2005). Briefly, all participants (*N*=9953) were asked to mail a stool sample to the study centre for laboratory analyses (the date of defecation and the date of receipt were documented) and to fill out a standardised questionnaire containing information on socio-demographic factors as well as detailed information regarding potential risk factors for various forms of chronic diseases, including CRC. In addition, medical data were recorded from the charts of patients' general practitioners. DNA was extracted from stool of all participants. In case the original stool sample contained more stool than the amount needed for DNA extraction, an additional sample of unprocessed stool was preserved and frozen until laboratory analysis, which was performed in March 2003. In the present analysis, all ESTHER participants for whom an additional sample of unprocessed stool was preserved were included (*N*=917). The study has been approved by local and state Ethics Committees.

### Laboratory analyses

For the quantitative measurement of tumour M2-PK, stool samples were thawed and a special stick capturing 4 mg of stool was loaded. Stool samples of both the CRC patients and the ESTHER participants were examined with the ScheBo® Tumour M2-PK test (ScheBo Biotech, Giessen, Germany), a sandwich enzyme-linked immunosorbent assay (ELISA) based on two monoclonal antibodies specific for tumour M2-PK. The test allows quantitative measurement of tumour M2-PK in 4 mg of stool with a lower detection limit of 2 U ml^−1^. All analyses were carried out in a central laboratory under standardised conditions.

### Statistical analyses

The CRC patients were characterised with respect to age, gender, body mass index, current smoking status, tumour stage, tumour location and neoadjuvant therapy. The subsample of the ESTHER study was described with respect to age, gender, body mass index, family history of CRC and smoking status; the distribution of levels of faecal tumour M2-PK among ESTHER participants was assessed according to these factors.

To estimate performance characteristics regarding the detection of CRC, the distribution of faecal tumour M2-PK concentrations among participants of the ESTHER Study and among CRC patients was compared. Sensitivity and specificity of the test were calculated at a cutoff value of 4 U ml^−1^, the cutoff level proposed by the manufacturer, and 95% confidence intervals were determined based on the exact binomial distribution. In addition, sensitivity and specificity were derived at a broad range of alternative cutoff values and receiver-operating characteristic curves (ROC) were constructed to visualise the discriminatory power of the test. To estimate the area under the ROC, the curve was linearly extrapolated beyond the lower detection limit of 2 U ml^−1^.

To assess the impact of different sample treatment between ESTHER study participants (stool samples were mailed before freezing) and CRC patients (stool samples were frozen immediately), sensitivity analyses were carried out based on the results of stability testing (Haug *et al*, 2006). For that purpose, faecal tumour M2-PK concentrations measured in ESTHER study participants were corrected according to the duration of sample mailing to estimate initial concentrations (the equation is given in Appendix A). The duration of sample mailing was calculated from the date of defecation and the date of receipt of the stool sample. On the basis of corrected values, ROC analyses were repeated as described above and compared to the ROC based on crude values.

## RESULTS

In Table 1, the CRC patients, the subgroup of the ESTHER participants included in this analysis and the whole ESTHER study population are described with respect to age, sex, body mass index, alcohol consumption, smoking status, family history of CRC (the latter information was available for ESTHER study participants only) and neoadjuvant treatment (CRC cases only). Median age among CRC patients was 65 years and about one-third of the patients were females. Median age in the subgroup of the ESTHER study was 62 years, a slight majority were females. The subgroup of the ESTHER study did not differ from the whole ESTHER study population with respect to the distribution of age, sex, body mass index, smoking status and family history of CRC.

**Figure 1:**
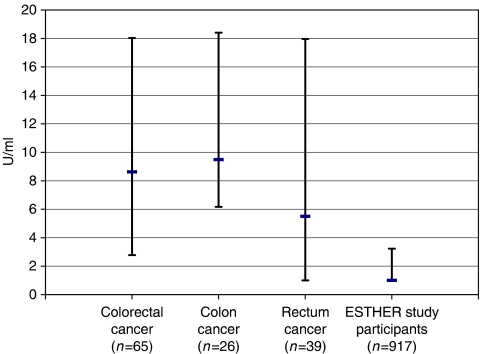
Median concentration and interquartile range of faecal tumour M2-pyruvate kinase concentrations among CRC patients and among ESTHER study participants.

The median (interquartile range) tumour M2-PK concentration for the CRC patients was 8.6 U ml^−1^ (2.8–18.0) with a striking difference between colon and rectum cancer (9.5 U ml^−1^, 6.2–18.4 and 5.5 U ml^−1^, <2–18.0, respectively). The median (interquartile range) tumour M2-PK concentration in the subsample of the ESTHER study was <2 U ml^−1^ (<2–3.2). The difference in median tumour M2-PK levels in stool between ESTHER participants and both subgroups of CRC patients was highly statistically significant (*P*<0.0001 for colon and rectum cancer, respectively; see Figure 1).

In the patient sample, 60% of tumours were located in the rectum (as the recruiting hospital was a referral centre for rectal cancer patients). The most frequent tumour stage was T3 (34 out of 65). Information allowing Dukes' classification was available for 48 patients. Twelve patients presented with Dukes' stage A, 18 with Dukes' stage B, 12 with Dukes' stage C and 6 with Dukes' stage D. At a cutoff value of 4 U ml^−1^, overall sensitivity (95% confidence interval) was 68% (55–79%), with a clear difference between colon cancer (85%) and rectum cancer (56%) (*P*=0.02; see Table 2). Sensitivity (95% confidence interval) by tumour size was 67% (22–96%), 44% (20–70%), 71% (53–85%) and 100% (40–100%) for T1–T4, respectively, and by Dukes' stage it was 67% (35–90%), 61% (36–83%), 67% (35–90%) and 100% (54–100%) for Dukes' stage A, B, C and D, respectively. As regards neoadjuvant therapy, no impact on sensitivity could be observed.

Of the ESTHER study participants, 21% showed a positive test result at the cutoff value of 4 U ml^−1^, that is, overall specificity was 79% (see Table 3). There was no clear association between age, sex, body mass index or family history of CRC and a positive test result. However, current smokers showed more frequently increased levels of tumour M2-PK in stool compared to never and former smokers (*P*=0.003).

**Figure 2:**
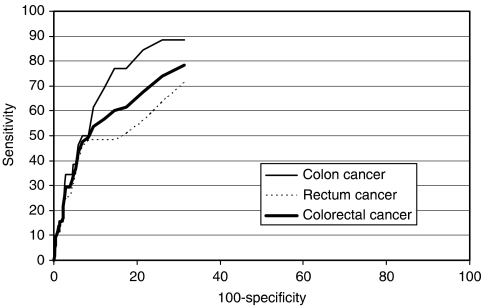
Receiver operating characteristics curve for the tumour M2-PK stool test in detecting patients with CRC.

The results of the ROC analysis are shown in Figure 2 for both colon and rectum cancer as well as for both forms of cancer combined. With an area under the curve (AUC) of 0.789, the overall discriminatory power of the test was good. The AUC for colon cancer (0.857) was larger than the AUC for rectum cancer (0.744).

Taking into account potential degradation of tumour M2-PK in samples from ESTHER study participants during mailing (stability testing for tumour M2-PK in stool showed relative concentrations of 73, 60, 49, 40 and 32% after 1, 2, 3, 4 and 5 days of storage at room temperature, respectively; Haug *et al*, 2006), decreased the estimate of specificity to 73% (70–76%) and shifted the ROC curve to the right, resulting in a slightly smaller AUC (0.736).

## DISCUSSION

In this study, performance characteristics for the tumour M2-PK stool test, a novel approach aimed at detecting CRC in the screening setting, were estimated by comparing tumour M2-PK concentrations in CRC patients and in a large sample of unselected older adults supposed to be healthy controls. With the cutoff level at 4 U ml^−1^ (recommended by the manufacturer), overall sensitivity for CRC was 68%. Albeit not perfect, this detection rate compares superior to FOBT and to the results reported for several DNA-based stool tests (Haug and Brenner, 2005a).

Colon cancer was detected with a remarkable sensitivity of 85%, which was higher than for rectum cancer (56%). Correcting for the unusually high proportion of rectal cancer patients in our sample (60% in our sample *vs* 20% in population-based series of CRC patients; Krebsregister Saarland, 2006) overall sensitivity for CRC would be 79%. This estimate is in line with the finding of a recently published prospective study reporting an overall sensitivity of 81% (Shastri *et al*, 2006). As regards sensitivity by tumour stage, no relevant difference could be observed between Dukes' A, Dukes' B and Dukes' C, but sensitivity was maximal for Dukes' D. However, given that in our study the number of cases per stage was rather limited, larger studies are needed to estimate sensitivity by tumour stage more precisely.

Specificity in terms of detecting CRC (ie, the denominator comprises subjects who do not have CRC) in this study was estimated to be about 79% (73% after correction for potential degradation) at the proposed cutoff level of 4 U ml^−1^. This estimate is based on tumour M2-PK concentrations determined in average-risk subjects who did not undergo colonoscopy, that is, the current status of the colon and rectum in this population sample serving as control group was unknown. The putative false positive rate may therefore comprise a small number of not yet diagnosed CRC patients. The prevalence of undiagnosed CRC in this age group has been reported to be below 1% (Imperiale *et al*, 2000; Segnan *et al*, 2002). Model calculations showed that specificity increases from 73.0 to 73.3% and 73.5% assuming a prevalence of undiagnosed CRC of 0, 0.5% and 1.0%, respectively (see Appendix B). Thus, the resulting error (ie, an underestimation of specificity) should be very small.

Another aspect that deserves more consideration is the potential presence of adenomas among controls with ‘false’-positive test result. Screening and autopsy studies found that the prevalence of adenomas in this age group is about 30% (Vatn and Stalsberg, 1982; Winawer *et al*, 1988; Rex *et al*, 1993) and other studies have reported a sensitivity of the tumour M2-PK test to detect adenomas of about 30% (Koss *et al*, 2005; Shastri *et al*, 2006). Model calculations showed that specificity increases from 73.0 to 74.7 and 79.0% assuming a sensitivity for adenomas of 30 and 40%, respectively (and assuming a prevalence of adenomas and CRC of 30 and 0.5%, respectively; see Appendix B). These figures suggest that specificity is likely to be slightly higher among subjects who are endoscopically proven free of colorectal lesions.

So far, specificity of the tumour M2-PK test has only been estimated based on selected high-risk controls (Hardt *et al*, 2004; Shastri *et al*, 2006). Our results suggest that specificity may be slightly higher in an asymptomatic screening population. Nevertheless, the false-positive rate of this test is still high and possibilities to enhance specificity should be explored. Considering the current specificity, it is debatable whether implementation of this test in a population-based screening setting is justifiable, from both an ethical and an economic point of view. However, while awaiting the development of an optimal screening test, it may be an option for individuals who wish a test with a higher sensitivity than the FOBT (provided that high sensitivity is confirmed by further studies) and who are well informed on possible false-positive findings. Furthermore, as previous simulations have shown (Haug and Brenner, 2005b), limited specificity in the long run may reduce CRC incidence by ensuring that a large proportion of screened subjects undergo colonoscopy even though more focused and economic approaches to achieve this goal would be highly desirable.

Apart from performance characteristics, this study was the first to determine faecal tumour M2-PK concentrations in a large sample of unselected older adults allowing investigation of potential determinants of this stool marker. The lack of association between important risk factors for CRC and increased levels of tumour M2-PK suggests that tumour M2-PK is not a marker of CRC risk, but the release of tumour M2-PK into the bowel is only increased when neoplastic lesions are already present. The observed association between current smoking status and higher levels of tumour M2-PK necessitates further examination. Given that the proportion of smokers was comparable among colon cancer patients, rectal cancer patients and ESTHER participants in our study, smoking status should not have confounded the estimates of sensitivity and specificity.

As regards more practical aspects, the analytic procedure of determining faecal tumour M2-PK is rather simple and inexpensive compared with other novel approaches, for example, DNA-based stool testing. Analyses are performed on only 4 mg of stool, and for sampling a special stick is used to capture the stool. On the one hand, this sampling modality appears to be less unpleasant than collecting a larger quantity of stool using a spatula or spoon, an aspect that may improve compliance with screening. On the other hand, given that the marker is determined quantitatively, the approach might be prone to variability owing to incorrect sampling. Considering the problem in a larger context, exact analyses might be a particular challenge of protein-based markers, where quantitative measurements are more common. In contrast, DNA-based tests predominantly provide qualitative (yes/no) answers.

In the interpretation of our results, the following limitations should be kept in mind. Samples from cases and controls could not be analysed in a blinded fashion. However, this may not have a relevant impact given the highly standardised and automated performance of this test. Another limitation concerns the collection of stool samples, which was different for cases and controls. To account for potential bias from this source, we performed sensitivity analyses based on the results from stability testing (Haug *et al*, 2006). These analyses showed that performance characteristics of the test only changed slightly when potential degradation during mailing of control samples was taken into account. The consistency of our findings with performance characteristics reported previously based on smaller sample sizes (Hardt *et al*, 2004; Shastri *et al*, 2006) further supports that the limitations of our study might not have distorted the results.

Given that previous studies on tumour M2-PK did not mention conditions of sample handling at all (Hardt *et al*, 2004; Shastri *et al*, 2006), the necessity of raising stability issues within our study is not only a limitation, but also a strength and allows further conclusion: Application of the test in a setting where a cold chain might be feasible would be expected to result in levels of specificity close to the corrected levels derived in this study and in levels of sensitivity close to those observed in this study. Conversely, application of the test in a setting where all stool samples are mailed without cooling would be expected to result in levels of specificity close to the uncorrected levels observed in this study and in levels of sensitivity that would be somewhat lower than observed in this study. The possibility of buffer-based stabilisation of faecal tumour-M2-PK should be explored.

In conclusion, regarding the comparatively high sensitivity of the tumour M2-PK stool test, its potential use for early detection of CRC and adenomas merits further investigation. At the current specificity, the test would result in a large proportion of follow-up colonoscopies. Possibilities to enhance specificity of the test (eg, by combination with other markers) need to be explored to make potential screening based on this test more focused and economic.

## Figures and Tables

**Table 1:** Sociodemographic, lifestyle and further characteristics of CRC cases, the subgroup of ESTHER study participants included in this analysis and the whole ESTHER study population

	**CRC patients**		**Subgroup of the ESTHER study *N* (%)**	**ESTHER study population (overall) *N* (%)**
	**Colon cancer *N* (%)**	**Rectal cancer *N* (%)**		
	***N*=26**	***N*=39**	***N*=917**	***N*=9953**
*Age group (years)*				
<50	4 (15)	3 (8)	0	
50–54	3 (12)	5 (13)	165 (18)	1697 (17)
55–59	3 (12)	3 (8)	150 (16)	1689 (17)
60–64	3 (12)	9 (23)	263 (29)	2704 (27)
65–69	6 (23)	8 (21)	194 (21)	2279 (23)
⩾70	7 (27)	11 (28)	142 (16)	1565 (16)
				
*Gender*				
Females	9 (35)	14 (36)	525 (57)	5469 (55)
Males	17 (65)	25 (64)	392 (43)	4484 (45)
				
*Body mass index (kg m* ^−*2*^ *)*				
⩽25	7 (27)	23 (61)	243 (27)	2706 (28)
>25–30	12 (46)	11 (29)	398 (44)	4566 (46)
>30	7 (27)	4 (11)	262 (29)	2564 (26)
				
*Current smoking status*				
Non-smoker	23 (88)	33 (87)	742 (83)	8053 (83)
Smoker	3 (12)	5 (13)	154 (17)	1652 (17)
				
*Family history of CRC*				
At least 1 first degree relative with CRC	NR	NR	68 (7)	904 (9)
				
*Neoadjuvant therapy*				
Chemotherapy	1 (4)	0	NA	NA
Radiotherapy	0	14 (36)	NA	NA
Both	1 (4)	1 (3)	NA	NA

Abbreviations: NA, not applicable; NR, not reported.

**Table 2:** Performance characteristics of the tumour M2-PK stool test at a cutoff value of 4 U ml^−1^ among patients with colorectal cancer

		**Sensitivity**	
**Patient group**	**Proportion of CRC cases tested positive**	**(%)**	**95% confidence interval**
*CRC*	44/65	68	55–79
Colon	22/26	85	65–96
Rectum	22/39	56	41–74
Proximal^a^	14/16	88	62–99
Distal^a^	30/49	61	46–75
T1^b^	4/6	67	22–96
T2^b^	7/16	44	20–70
T3^b^	24/34	71	53–85
T4^b^	4/4	100	40–100
Dukes' A	8/12	67	35–90
Dukes' B	11/18	61	36–83
Dukes' C	8/12	67	35–90
Dukes' D	6/6	100	54–100
			
*Neoadjuvant therapy*			
No	33/48	69	54–81
Yes	11/16	69	41–89

Abbreviations: CRC=colorectal cancer.

a Proximal / distal to the splenic flexure.

b T stage according to the TNM classification.

**Table 3:** Distribution of subjects in the population sample (ESTHER study participants), and proportion of positive test results for faecal tumor M2-PK at a cutoff value of 4 U ml^−1^ according to age, sex, body mass index, cigarette smoking status and family history of colorectal cancer

	**Proportion of ESTHER participants tested positive**		***P*-value (*χ*^2^ test)**	**Specificity (95% CI)**
	196/917	21.4%		78.6% (75.8–81.2%)
Age group (years)				
50–54	30/165	18.2%		
55–59	34/150	22.7%		
60–64	56/263	21.3%		
65–69	42/194	21.6%		
70–75	34/142	23.9%	0.789	
Sex				
Females	104/525	19.8%		
Males	92/392	23.5%	0.181	
				
Body mass index (kg m^−2^)				
⩽25	45/243	18.5%		
>25–30	78/398	19.6%		
>30	67/262	25.6%	0.097	
				
*Cigarette smoking status*				
Never smoker	98/484	20.2%		
Former smoker	45/258	17.4%		
Current smoker	48/154	31.2%	0.003	
				
*Family history of CRC*				
First degree relative with CRC	13/68	19.1%		
No first degree relative with CRC	183/849	21.6%	0.637	

## Appendix A

The following equation was used to estimate initial tumour M2-PK concentrations among ESTHER study participants: Con_0_=Con_*i*_/relcon_i_, where

Con_0_ denotes the initial concentration, Con_*i*_ denotes the concentration measured after *i* days of (uncooled) sample mailing and relcon_i_ denotes the relative concentration after *i* days of storage at room temperature as determined in a preceding stability testing study (Haug *et al*, 2006).

## Appendix B

To refine the estimated specificity of the tumour M2-PK test, the following components were first calculated:

No. of controls testing positive: *N*_pos_=*N*^*^*P*_pos_

No. of controls with undiagnosed CRC: *N*_crc_=*N*^*^*P*_crc_

No. of controls with adenomas: *N*_ad_=*N*^*^*P*_ad_

No. of controls with undiagnosed CRC testing positive: *N*_crc_pos__ = *N*_crc_^*^ SE_crc_

No. of controls with adenomas testing positive: *N*_ad_pos__ = *N*_ad_^*^SE_ad_

where, *N* denotes the whole number of controls, *P*_pos_ the proportion of controls testing positive (positivity rate), *P*_crc_ and P_ad_ denote the prevalence of undiagnosed CRC and adenomas among controls, respectively, and SE_crc_ and SE_ad_ denote the sensitivity of the test to detect CRC and adenomas, respectively.

Inserting these components, specificity in terms of detecting CRC (SP_crc_, ie, healthy means not having CRC) and specificity in terms of detecting adenomas (SP_ad_, ie, healthy means having neither CRC nor adenomas) were calculated as follows:

MN Wente and CM Seiler are members of the clinical study centre at the department of General, Visceral and Trauma Surgery, University of Heidelberg, which received an institutional grant of 3000 Euro from ScheBo Biotech to perform this study.
